# Sense of Coherence and Past and/or Present Mental Health Problems Among Conscripts at Military Call-up in Northern Finland

**DOI:** 10.1093/milmed/usae570

**Published:** 2025-01-09

**Authors:** Carlos Sirkiä, Heli Koivumaa-Honkanen, Kai Parkkola, Tuula Hurtig

**Affiliations:** Research Unit of Clinical Medicine, Psychiatry, University of Oulu, Oulu, 90014, Finland; Faculty of Education and Psychology, University of Oulu, Oulu, 90014, Finland; Institute of Clinical Medicine, Psychiatry, University of Eastern Finland, Kuopio, 70210, Finland; Faculty of Medicine and Health Technology, Tampere University, Tampere, 33014, Finland; National Defence University, Helsinki, 00861, Finland; Research Unit of Clinical Medicine, Psychiatry, University of Oulu, Oulu, 90014, Finland; Medical Research Center, University of Oulu, Oulu University Hospital, Oulu, 90220, Finland

## Abstract

**Introduction:**

Sense of coherence (SOC) refers to the psychosocial aspects and origins of health. Sense of coherence is related to physical and psychological health and quality of life. Military studies on SOC are commonly related to military deployment or operations, military training, and military fitness. Sense of coherence is assessed using a self-report scale. The total score of the scale indicates the level of SOC. Psychometric research studies over 2 past decades, however, suggest that the SOC scale is a multidimensional measure, and the latent factors should be considered as separate dimensions of SOC. Thus far, there are no previous factorial structure studies of the SOC scale in military contexts or in military populations. The dimensions of SOC have not been investigated in relation to mental health and subjective well-being of soldiers, military personnel, or military recruits/conscripts.

**Materials and Methods:**

This study examined the structure of the self-report SOC scale among 2614 military conscripts at military call-up (before obligatory military service) in Northern Finland. Confirmatory factor analysis was used to test 4 different structure models based on previous studies of the SOC scale (1-, 2-, or 3-factor models). The dimensions of SOC were investigated in relation to conscripts’ self-reported past and/or present mental health problems. Demographic variables were explored. Nonparametric tests for group and pairwise analyses were used.

**Results:**

The 11-item 2-factor model fitted the data best. The 2-factor structure represented the cognitive behavioral (comprehensibility and manageability) and motivational (meaningfulness) dimensions of SOC. The motivational dimension was higher among conscripts who reported past but no present mental health problems as compared to conscripts who reported past and present mental health problems. A similar difference was not found in the cognitive behavioral dimension. Both cognitive behavioral and motivational dimensions of SOC were higher among conscripts who reported not having past or present mental health problems.

**Conclusions:**

SOC among conscripts is a 2-dimensional model. The experience of recovery from past mental health problems is related to a stronger motivational dimension (meaningfulness towards life), which shows upon obligatory military service and training. Sense of coherence motivational aspect may be useful when training recruits. Demographic variables, such as economic situation, family structure, and parents’ employment, should be considered when assessing past and/or present mental health problems and SOC.

## INTRODUCTION

Sense of coherence (SOC) is a theoretical construct and the cornerstone of American Israeli medical sociologist Aaron Antonovsky’s salutogenic model of health. The model refers to the origins of health^1,2^ and focuses on resources for maintaining health and health-promoting processes.^3–5^ Sense of coherence refers to a global orientation to one’s inner and outer environments, which is hypothesized to be a significant determinant of location and movement on the health ease/disease continuum.^6^

In its original definition, SOC “is a global orientation that expresses the extent to which one has a pervasive, enduring though dynamic feeling of confidence that (1) the stimuli deriving from one’s internal and external environments in the course of living are structured, predictable, and explicable; (2) the resources are available to one to meet the demands posed by these stimuli; and (3) these demands are challenges, worthy of investment, and engagement.”^2^ These 3 components are referred as (1) comprehensibility, (2) manageability, and (3) meaningfulness.^2,6^ With more contemporary terms, the components can be referred to as cognitive (comprehensibility), instrumental/behavioral (manageability), and motivational (meaningfulness) dimensions.^7^

To operationalize the concept of SOC and its dimensions, Antonovsky developed a self-report measure that consists of 29 verbal statements (SOC-29) and covers the 3 theoretical components.^2^ Later, derived from SOC-29, he introduced a shortened 13-item version of the scale (SOC-13).^5^ Since their development, both scales have been widely used, and the scales have translations to at least 49 languages.^3,8^ Their internal consistency has been found to be acceptable/good, with Cronbach’s alpha ranging between 0.70 and 0.95.^3^ The structural (construct) validity, however, is more complex. Some studies have reported a unidimensional 1-factor structure, whereas, in other studies, a multidimensional 2-factor model or, most commonly, a 3-factor model has been proposed.^7^ The structure has varied depending on the scale used, as well as, on the population studied. At the time of scale and theory development, Antonovsky maintained the components of SOC on theoretical grounds only, and not until later research, the focus has been on construct validity in more depth.

Empirical research has shown that SOC is associated with health as the salutogenic model postulates. Based on systematic reviews and meta-analyses, SOC is related to perceived health,^4^ especially to mental health,^9^ posttraumatic stress,^10^ physical activity,^11^ and quality of life.^12^ In addition to general populations, SOC has been explored in military contexts as well. Most of the research studies concern soldiers’ psychological well-being, especially related to military deployment or operations. For example, Wesemann et al.^13^ reported the development of a screening instrument for assessing psychological fitness to deployment of German Armed Forces combat soldiers in Afghanistan. Self-report questionnaires about resilience, SOC, quality of life, mental disorders, and post-traumatic growth were used. Low scores on the social relationship and psychological scales and high scores on the somatoform and stress scales were positively correlated with low psychological fitness at pre- and post-deployment. Bäccman et al.^14^ studied how the members of military personnel in a Swedish Naval Force were affected by deployment in a counter-piracy operation off the coast of Somalia. Resilience and psychological well-being improved during deployment, including SOC. Wallenius et al.^15^ examined self-reported reactions and performance when facing risks and dangers on peacekeeping missions among Swedish military observers in Yugoslavia, Angola, Georgia, Kashmir, the Middle East, and Mozambique. Cognitive limitations in danger incidents were related to complicating situational factors and high individual vulnerability factors, such as low SOC.

Military training and SOC have also been the focus of interest. For example, Vaara et al.^16^ studied how physical fitness, body composition, and hormonal variables together with self-reported psychological factors, such as SOC, predicted dropout from a 10-day intense winter military survival training in Finland. Most of the physiological and psychological variables at the baseline did not predict dropout, but baseline aerobic fitness and serum cortisol were associated with dropout. Larsen and Leboeuf-Yde^17^ studied how baseline SOC could predict challenges in physical fitness and its indicators, such as low back pain and associated leg pain, in Danish military recruits subjected to the first 3 months of military service (i.e., training). Low SOC successfully predicted leg pain, but only poorly low back pain.

Finally, there are a few cross-sectional population-based epidemiological studies of SOC in military populations. Kronström et al.^18^ studied Finnish 18-year-old males attending obligatory military call-up in 1999 and 2009 on time-trend changes in psychosocial well-being, psychopathology, substance use, suicidality, bullying, and SOC. The prevalence of minor mental health problems before military service decreased in 10 years, whereas severe mental health problems remained stable. Attention problems and somatic complaints increased, while SOC remained stable. Stable SOC indicated resilience during the 10-year follow-up period. In another study from Finland, Ristkari et al.^19^ reported that among Finnish male conscripts, SOC correlated strongly with young men’s perceived mental health problems, diagnosed psychiatric disorders present at military call-up health examinations, and utilization of mental health services. Mental health breakdown and risk behavior, such as substance use and suicidal tendencies, were associated with low SOC. In turn, Giotakos^20^ studied the comorbidity of suicidal behavior and substance use among Greek male conscripts and the association between personal coping resources and the severity of these behaviors. All the subgroups with suicidal ideation or behavior showed a significantly lower SOC as compared to conscripts without suicidal ideation or behavior. Conscripts with past or current substance use had a significantly higher incidence of past or current suicidal ideation or behavior, as compared with those without a history of substance use.

Military population and military environment-related SOC research is most often based on 13-item self-report scale,^6^ which has been used and validated in several empirical studies.^3^ Following Antonovsky’s original guidance, only the total score of the scale is applied when assessing the level of SOC. However, contemporary evidence suggests that Antonovsky’s SOC scale may not be a unidimensional but rather multidimensional measure of SOC consisting of at least 2 or 3 latent factors.^3,20^ The dimensions (factor solutions) of the SOC scale have also been related to the number of items included in the scale. Statistical analyses have shown that some of the items seem to have completely distinct factor loadings, unlike what was theoretically intended. Because of this problematic nature of some of the items, shorter than 13-item versions of the SOC scale exist as well. Psychometric analyses support the exclusion of some of the items altogether.^21,22^

Previous studies have shown support for the validity of 9-item^23–25^ and 11-item^26^ version of the Antonovsky’s SOC-13 scale. The 11-item version excludes psychometrically the most problematic 2 items of the 13-item SOC, namely item 2 (“Has it happened in the past that you were surprised by the behavior of people whom you thought you knew well?”) and item 3 (“Has it happened that people whom you counted on disappointed you?”). The 9-item version includes the 3 highest loading items on each of the 3 components (i.e., comprehensibility, manageability, and meaningfulness) and excludes items with the weakest loadings in Finnish and Norwegian^23,24^ or Czech^25^ adult population. In addition to items 2 and 3, the 9-item version excludes item 11 (“When something happened, have you generally found that: you overestimated or underestimated its importance – you saw things in the right proportion”) and item 4^23,24^ (“Until now your life has had: no clear goals or purpose at all—very clear goals and purpose”) or item 1^25^ (“Do you have the feeling that you really don’t care about what is going on around you?”).

The dimensions of SOC among military population have remained unknown because of the lack of any factorial structure studies in military contexts. Research studies over three past decades have shown that the structure of the SOC is more complex than originally proposed, and the construct is a multidimensional rather than a unidimensional measure.^21^ The multidimensional measure refers most commonly to the 3-factor model with the SOC components of comprehensibility, manageability, and meaningfulness as separate factors, or to the 2-factor model with SOC components of comprehensibility and manageability as a combined factor and meaningfulness as a separate factor. Given the evidence of multifactorial structure, it is reasonable to assume the multidimensionality of SOC in military populations and military contexts as well. Research to confirm this is, however, highly needed.

### Aims of the Study

This study had 2 goals. The first aim was to investigate the level of SOC in relation to conscripts’ self-reported mental health problems. The goal was to find out whether there are differences in the dimensions of SOC among conscripts with/without past and/or mental health problems among a large and representative sample of military conscripts at military call-up (i.e., before obligatory military service and training) in Finland. The second aim was to investigate the structure of the self-reported SOC scale. The goal was to determine whether the SOC scale in military call-up is a rather unidimensional (1-factor) or multidimensional (3-factor or 2-factor) measure.

## MATERIALS AND METHODS

The participants for this study consisted of 2614 male conscripts who attended obligatory military call-ups and voluntarily filled out the study questionnaire. The study sample covered 57.4% of all the conscripts attending military call-ups in Northern Finland in 2014. Military call-up in Finland is based on general conscription, meaning all Finnish male citizens become liable for participating in the military call-up event when they turn 18 years. At military call-up, young men receive information on the conscript service, and their suitability (i.e., fitness for military service) is assessed and determined. The service period and service location are assigned, or possible exemption from military service is decided. Military call-ups are organized annually from August to December, and the call-ups are based on the Conscription Act by the Ministry of Defence in Finland. More detailed description of the military call-up system, Finnish Defence Forces, and military training in Finland is presented in our previous, recently published study.^27^

The study questionnaire included sociodemographic and background factors, life history experiences, and validated measures of psychosocial health and subjective well-being. The participants gave their written consent to use the data in scientific research. The decision to participate (or not to participate) in the study did not affect the participant’s recruitment in military service and/or training. The study received a statement from the Ethical Committee of the Northern Ostrobothnia Hospital District. Defence Command Finland gained research permissions from the year 2014 onwards (AK8233, AN11380, AQ24309, AU7729, AU12646)

### Self-report of Mental Health Problems

The study questionnaire included 2 questions, 1 for past and 1 for present mental health problems: (1) “Have you ever been diagnosed with a mental health problem?” and (2) “Do you have a mental health problem at the moment?”. The questions were followed by a description of the perceived mental health problem if any had occurred. Participants were asked to freely describe which was the mental health problem (“What mental health problem have you had previously?” or “What mental health problem you have at the moment?”).

There were altogether 107 conscripts (4.1%) who reported having mental health problems. Based on participants’ descriptions, mental health problem(s) concerned most often mood and/or anxiety disorders (67.5%), neuropsychiatric disorders (e.g., attention-deficit/hyperactivity disorder, autism spectrum disorder) (15.1%), and social or behavioral problems (e.g., social phobia, impulsive behavior) (4.6%). Severe mental health problems (e.g., psychosis, trauma, dissociative, or personality disorder) were in some cases also reported (7.0%). The description of mental health problems was based on conscripts’ self-report and no actual medical or psychiatric evaluation at this point was performed.

Conscripts were divided into 4 mental health groups: (1) conscripts reporting no past and no present mental health problems (*n* = 2446), (2) conscripts reporting past but no present mental health problems (*n* = 36), (3) conscripts reporting no past but present mental health problems (*n* = 24), and (4) conscripts reporting present and past mental health problems (*n* = 47). These 4 groups were the basis for group and pairwise comparisons investigating the level and dimensions of SOC as well as demographic characteristics among conscripts. Of all the participants (*N* = 2614), 61 conscripts (2.3 %) had not reported any information regarding mental health problems, and these were addressed as missing data.

### SOC Scale

SOC was evaluated using the 13-item SOC scale.^6^ Each item is scored on a 7-point Likert scale from 1 to 7. The total score of SOC-13 ranges from 13 to 91. A high score expresses strong SOC, and a low score expresses weak SOC. Based on theoretical grounds, there are 5 items of comprehensibility (items 2, 6, 8, 9, and 11), 4 items of manageability (items 3, 5, 10, and 13), and 4 items of meaningfulness (items 1, 4, 7, and 12).^6^ The verbal statements of SOC-13 are presented in **Table 1**. The answering options/phrases and more detailed scoring in each item are presented in the original study of the scale.^6^

**Table 1.** The verbal statements (items) of the short version of the SOC questionnaire (SOC-13).

No. of item in SOC-13	Verbal statement^a^	Component^a^
1.	Do you have the feeling that you really don’t care about what is going on around you?	ME
2.	Has it happened in the past that you were surprised by the behavior of people whom you thought you knew well?	CO
3.	Has it happened that people whom you counted on disappointed you?	MA
4.	Until now your life has had: “no clear goals or purpose at all”—“very clear goals and purpose”	ME
5.	Do you have the feeling that you are being treated unfairly?	MA
6.	Do you have the feeling that you are in an unfamiliar situation and don’t know what to do?	CO
7.	Doing the things you do every day is: “a source of deep pleasure and satisfaction”—“a source of pain and boredom”	ME
8.	Do you have very mixed-up feelings and ideas?	CO
9.	Does it happen that you have feelings inside you would rather not feel?	CO
10.	Many people, even those with a strong character, sometimes feel like sad sacks (losers) in certain situations. How often have you felt this way in the past?	MA
11.	When something happened, have you generally found that: “You overestimated or underestimated its importance?”—“You saw things in the right proportion?”	CO
12.	How often do you have the feeling that there is little meaning in the things you do in your daily life?	ME
13.	How often do you have feelings that you are not sure you can keep under control?	MA

Abbreviations: CO, comprehensibility; MA, manageability; ME, meaningfulness; SOC, sense of coherence.

a As presented in the original report of the scale^6^.

Missing item-level data of the SOC scale were replaced by the mean score of a particular item. There were 107 cases in which the answer to only 1 item was missing, 43 cases in which the answers to 2–12 items were missing, and 72 cases in which the answers to all 13 items were missing. Altogether, there were 222 cases (8.5%) with missing answers to the SOC scale.

### Statistical Analyses

All descriptive statistics, variance testing, and pairwise analyses were carried out using IBM SPSS software version 29.0. To choose the appropriate statistical methods, tests of normality were conducted. The distribution of the SOC scale total score departed significantly from normality (*W* = 0.98, *P* < .01) as did the distribution of comprehensibility (*W* = 0.97, *P* < .01), manageability (*W* = 0.96, *P* < .01), and meaningfulness scores (*W* = 0.97, *P* < .01). This led to using nonparametric tests throughout the statistical analyses. The Kruskal–Wallis test (i.e., the nonparametric equivalent of the one-way ANOVA) was used to examine the difference between the medians of groups consisting of conscripts reporting mental health problems. The Mann–Whitney *U* test (i.e., the nonparametric equivalent to the 2-sample independent *t*-test) was used to examine the difference between ranking values of pairwise group comparisons. Nonparametric tests are based on ranked orders instead of actual scale scores. Multiple testing correction (i.e., Bonferroni correction) was applied in all analyses.

### Confirmatory Factor Analysis

The structure of the SOC scale was investigated with confirmatory factor analysis (CFA) using Mplus software version 8.4. Confirmatory factor analysis was based on the inspection of the most common model fit statistics: comparative fit index (CFI), Tucker–Lewis index (TLI), root mean square error of approximation (RMSEA), and standardized root mean square residual (SRMR). The accepted model fit included CFI and TLI values above 0.90, and RMSEA and SRMR values below 0.08. The preferred (stricter) model fit criteria included RMSEA close to 0.60 and CFI/TLI close to 0.95. The selected cut-off values were based on statistical literature.^28,29^ All CFA models were estimated using the full information maximum likelihood estimation method with robust standard errors, which can effectively handle missing at random data as well as departures from normality which was the case in our data, as described earlier. The models to be confirmed were selected from previous structure studies of the 13-item SOC scale with healthy (nonclinical) adult populations.^6,23–26^ The selected models included 1-, 2-, or 3-factor solutions.

## RESULTS

The demographic characteristics of the study population are presented in **Table 2**. Between the mental health groups, there was a statistically significant difference in family structure (χ2 (3) = 28.80, *P* < .001), economic situation (χ2 (3) = 22.65, *P* < .001), and parents’ employment (χ2 (3) = 10.50, *P* < .05), whereas in demographic factors of education, relationship status, living situation, or parents’ education, statistically significant differences were not found. Conscripts with no past and no present mental health problems had more likely a family structure with both parents living together as compared to conscripts with present and past mental health problems (*U* = 39,563.00, *P* < .001). Conscripts with no past and no present mental health problems were also more likely to have economic situation as good as compared to conscripts with present but no past mental health problems (*U* = 19,051.50, *P* < .001) and conscripts with present and past mental health problems (*U* = 43,489.50, *P* < .01). Conscripts with present and past mental health problems had more likely parents’ employment as both unemployed as compared to conscripts with no past and no present mental health problems (*U* = 46,457.00, *P* < .001). Otherwise, no statistical differences between the groups were found.

**Table 2.** Characteristics of the study population (*N* = 2614).

Charateristic		%
Year of birth^a^	1996	95.5%
	1995 or earlier	4.5%
Native language	Finnish	99.1%
	Other	0.9%
Education^b^	No high school diploma	10.6%
	High school diploma, vocational school	48.5%
	High school diploma, high school	40.9%
Relationship status	In a relationship	27.9%
	Not in a relationship	72.1%
Family structure	Parents living together	71.5%
	Parents not living together^c^	28.5%
Living situation	Living with parents	79.7%
	Not living with parents	20.3%
Economic situation^d^	Good	45.9%
	Moderate or poor	53.4%
Parents’ education^e^	Primary and lower secondary education	1.6%
	High school diploma^f^	46.3%
	Polytechnics or university degree	52.3%
Parents’ employment status^g^	Both employed	67.5%
	Only mother/father employed	24.5%
	Both unemployed	4.8%

a The mean age was 18.1 years (SD = 0.4).

b Completed or ongoing education.

c Parents are divorced or have never been living together.

d Based on subjective estimation.

e The highest education of mother and/or father.

f High school diploma in vocational school or in high school.

g The employment status of mother and/or father.

The CFA model fit statistics for 1-factor, 2-factor, and 3-factor models in 13-item, 11-item, and 9-item versions of the SOC scale are presented in **Table 3**. Based on model fit statistics, the 1-factor, 2-factor, or 3-factor model of the 13-item scale could not be accepted. Similarly, the 1-factor model of 11-item and 9-item scales was not acceptable. Acceptable model fit indices were found in the 2-factor and 3-factor models of 11-item and 9-item versions of the scale. However, the 3-factor models had notably high factor covariance (i.e., intercorrelations between the latent factors) leading to the preference of 2-factor models. Of these models, the 11-item version had a more suitable model fit (i.e., lower RMSEA and SRMR, higher CFI and TLI) than the 9-item version. Therefore, CFA indicated that the 11-item 2-factor model had best fit, and this version was selected as a suitable SOC measure in the military call-up population representing the level of SOC with 2 distinct dimensions: factor 1 (cognitive behavioral) and factor 2 (motivational).

**Table 3.** The model fit statistics for the alternative factor structure models of the SOC scale among male conscripts in Finland (*N* = 2614).

	χ^2^	df	RMSEA	CFI	TLI	SRMR
13-item 1-factor model	1488.58^***^	65	0.093	0.815	0.779	**0.064**
13-item 2-factor model	1274.20^***^	64	0.086	0.843	0.809	**0.059**
13-item 3-factor model	1265.04^***^	62	0.087	0.844	0.804	**0.059**
11-item 1-factor model (excluding items 2 and 3)	649.84^***^	44	**0.074**	**0.901**	0.876	**0.047**
11-item 2-factor model (excluding items 2 and 3)	435.00^***^	43	**0.060**	**0.936**	**0.918**	**0.039**
11-item 3-factor model (excluding items 2 and 3)	414.50^***^	41	**0.060**	**0.936**	**0.918**	**0.038**
9-item 1-factor model (excluding items 2, 3, 4, and 11)	548.71^***^	27	0.087	0.894	0.859	**0.052**
9-item 2-factor model (excluding items 2, 3, 4, and 11)	364.52^***^	26	**0.072**	**0.931**	**0.905**	**0.042**
9-item 3-factor model (excluding items 2, 3, 4, and 11)	338.91^***^	24	**0.072**	**0.936**	**0.904**	**0.041**
9-item 1-factor model (excluding items 1, 2, 3, and 11)	456.69^***^	27	**0.079**	**0.914**	0.885	**0.046**
9-item 2-factor model (excluding items 1, 2, 3, and 11)	341.26^***^	26	**0.069**	**0.937**	**0.912**	**0.040**
9-item 3-factor model (excluding items 1, 2, 3, and 11)	318.93^***^	24	**0.070**	**0.941**	**0.911**	**0.039**

*** *P* < .001. Abbreviations: CFI, comparative fit index; RMSEA, root mean square error of approximation; SOC, sense of coherence; SRMR, standardized root mean square residual; TLI, Tucker–Lewis index.

Acceptable model fit values are bolded.

**Figure 1.**
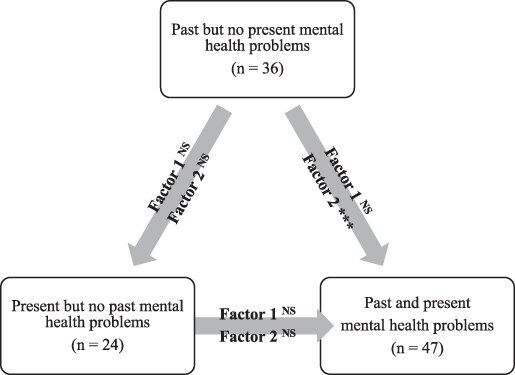
Pairwise comparisons of SOC-11 factor ranking values among groups of the conscripts reporting mental health problems at military call-up (*N* = 107). ***P = .001. Abbreviation: NS, not significant. Factor 1 = cognitive behavioral dimension of SOC, factor 2 = motivational dimension of SOC. All *P*-values have been Bonferroni corrected. The direction of arrow points the ranking value from higher to lower using the Mann–Whitney *U* test (i.e., nonparametric equivalent to the 2-sample independent *t*-test).

The mean of SOC-11 total score among the whole study population was 57.01 (SD = 10.08), mean average score being 5.18 (SD = 0.92). The mean of SOC-11 factor 1 score was 36.22 (SD = 7.00), mean average score being 5.17 (SD = 1.00). The mean of SOC-11 factor 2 score was 20.78 (SD = 7.00), mean average score being 5.19 (SD = 1.03). The means and standard deviations of SOC-11 total score and 2-factor scores in the groups of conscripts having past and/or present mental health problems are presented in **Table 4**. Between the mental health groups, there was a statistically significant difference in SOC-11 total (χ^2^ (3) = 108.52, *P* < .001), SOC-11 factor 1 (χ^2^ (3) = 88.77, *P* < .001), and SOC-11 factor 2 (χ^2^ (3) = 100.85, *P* < .001). Conscripts with no past and no present mental health problems had higher ranking values in SOC-11 total and in both factors as compared to all other groups (*P* < .05). Regarding the conscripts with past and/or present mental health problems, the pairwise comparisons of SOC-11 factor 1 and factor 2 scores are presented in Figure 1.

**Table 4.** Means (*M*) and standard deviations (SD) of SOC scale (SOC-11) average score and 2-factor average scores among conscripts reporting past and/or present mental health problems.

	No past and no present mental health problems (*n* = 2446)		Past but no present mental health problems (*n* = 36)		Present but no past mental health problems (*n* = 24)		Present and past mental health problems (*n* = 47)	
	*M*	SD	*M*	SD	*M*	SD	*M*	SD
SOC-11 total	5.24	0.87	4.70	1.10	3.98	0.94	3.66	1.06
SOC-11 factor 1	5.23	0.96	4.73	0.98	3.96	0.99	3.81	1.19
SOC-11 factor 2	5.25	0.97	4.67	1.46	4.01	1.17	3.50	1.24

Factor 1 = cognitive behavioral dimension of SOC, factor 2 = motivational dimension of SOC.

Abbreviation: SOC, sense of coherence.

## DISCUSSION

This study examined SOC and its relation to past and/or present mental health problems as well as the structure of the self-reported SOC scale among male conscripts in Northern Finland. Confirmatory factor analysis revealed acceptable fit only in the 2-factor and 3-factor models of the 11-item and 9-item scales. In the 3-factor models, the covariance between factors was notably high, leading to the preference of 2-dimensional models. When comparing the model fit statistics between the 2-factor models of 9-item and 11-item SOC scales, the fit statistics were slightly better in the 11-item scale (i.e., lower RMSEA and SRMR, higher CFI and TLI). Also, when using stricter CFA model fit criteria with RMSEA close to 0.60 and CFI/TLI close to 0.95,^29^ the 11-item 2-factor model was the only acceptable to represent the multidimensional SOC among conscript military call-up population.

The 2 factors embody the cognitive behavioral (comprehensibility and manageability) and motivational (meaningfulness) dimensions of SOC. This factor structure is in line with contemporary psychometric research of the scale.^30^ In contrast to the recent report of the 9-item scale with an 18–86-year-old adult population,^25^ our study did not find support for the 1-factor model, suggesting that among 17–18-year-old conscripts, the multidimensionality of SOC holds even when using the systematically shortened scale.

As expected, conscripts without mental health problems had stronger SOC dimensions as compared to those with these problems. A new finding is that conscripts with only past mental health problems had stronger motivational dimension of SOC as compared to conscripts with both past and present mental health problems. Similar difference was not found in the cognitive behavioral dimension of SOC. Also, there were no statistical differences in the 2 SOC dimensions between conscripts with only present mental health problems and conscripts with both past and present mental health problems. Thus, the motivational dimension of SOC seems to be distinguishable, especially among those conscripts who have recovered from mental health problems. Although research supports the notion that having a feeling of purpose in life helps people rise above mental health issues,^31^ this is the first time, to our knowledge, that the association was found among military conscripts and using SOC measure.

Regarding SOC and mental health, demographic factors should be taken closely into account. Our study sample consisted of young men, the majority being at the age of 17 or 18 years. Previous research has shown that sociodemographic variables and socioeconomic status might play an important role in SOC during the adolescent years.^32^ For example, higher levels of parents’ education,^33^ higher economic status,^34^ and living with 2 parents^35^ have been important indicators of stronger SOC. Similarly, parental divorce or serious illness or death of parents have indicated weaker SOC among young men.^36^ We found that economic situation, family structure, and parents’ employment varied depending on past and/or mental health problems, as did SOC in some parts, as described earlier. However, these and other demographic characteristics in relation to SOC should be explored in more depth in future studies, preferably with statistical modeling.

### Strengths and Limitations

The strength of this study is that it has a large and representative study population of male conscripts in Northern Finland. Given Finland’s arctic environment and recent membership in NATO, this study widens the knowledge of psychosocial aspects of conscripts entering military service and training in northern circumstances. Another strength is using a broadly validated self-report measure that allows taking into account cumulative empirical research from more than 3 decades and various study settings. The limitations of this study include a cross-sectional study design only with no possibility to conduct any follow-up during the actual military service. As the SOC scores did not follow normal distribution and the sample sizes of group comparisons stayed rather small (<50), the statistical analyses were based on nonparametric tests only. Whereas nonparametric tests are more robust and conservative, parametric tests would yield more statistical power. Also, mean imputation may be limited when one does not have a normal distribution. Therefore, studies with larger sample sizes, especially regarding the groups of past and/or present mental health problems, are needed. Another methodological limitation of our study could be that the assessment of the psychosocial aspect of health and well-being (i.e., SOC) as well as mental health problems were based solely on self-report methods. Considering that self-report data are dependent on individuals’ perceptions and interpretations, data may be subject to response bias. Future studies should encompass more thoroughly actual medical diagnoses as part of mental health evaluation.

Paying attention to strengths and limitations, our study suggests that the occurrence of mental health problems is related to different dimensions of SOC before military service and training. In particular, the motivational dimension may become potentially applicable to military personnel such as leaders and trainers. There are indications of the importance of motivation in military training and performance. For example, in a U.S. study, significant prediction on the special forces soldiers’ performance was based on motivational attributes during training and implications for incorporating motivational measures into the special forces selection.^37^ In another U.S. study, peer rankings predicted special forces training outcomes better than military personnel ratings as peers placed more importance on motivation and interpersonal performance.^38^ The results from our study and the abbreviated 11-item SOC scale, especially its meaningfulness (motivational) dimension, might give new interesting insights into military training and recruitment.

## CONCLUSION

Self-report SOC scale (SOC-11) at military call-up consists of cognitive behavioral (comprehensibility–manageability) and motivational (meaningfulness) dimensions of SOC. Recovery from past mental health problems is related to stronger motivational dimension, i.e., meaningfulness toward life, which shows upon obligatory military service.

## Data Availability

The data underlying this article were provided by the Finnish Defence Force by permissions (AK8233 and AU12646). The data cannot be shared publicly due to the terms of the permission and due to the privacy of individuals who participated in the study.

## INSTITUTIONAL CLEARANCE

Institutional clearance does not apply.
